# Care pathways and healthcare management in a COVID-19 triage psychiatric inpatient ward at south london and maudsley nhs foundation trust

**DOI:** 10.1192/j.eurpsy.2021.812

**Published:** 2021-08-13

**Authors:** L. Rebolledo-Ojeda, J. Tweed, R. Williams, J. Aygeman, O. Khalid, M. Pinto Da Costa

**Affiliations:** Virginia Woolf Ward, South London and the Maudsley NHS Trust, London, United Kingdom

## Abstract

**Introduction:**

The COVID-19 pandemic has enforced the restructuring of inpatient psychiatric services. In the UK, the South London and Maudsley NHS Foundation Trust has introduced a triage ward system to ensure all patients have a COVID test prior to admission to the general ward with the aim to reduce COVID transmission amongst psychiatric inpatients.

**Objectives:**

To characterise the flow of patients through a COVID-19 psychiatric triage ward and the protocol of assessment and management used.

**Methods:**

Descriptive analysis of patients admitted to a COVID-19 triage ward since its creation.

**Results:**

The caseload of patients admitted to the COVID-19 triage ward since its inception will be presented. This will include the profile of patients admitted, their status (formal/informal) and their acceptance of COVID-19 tests. The protocol followed at this COVID-19 triage ward will be presented, and the challenges faced and suggestions to overcome them will be discussed.

**Conclusions:**

This presentation aims to share the workflow and protocols adopted at a COVID-19 triage ward in the UK, discussing challenges experienced as well as good practices.

**Conflict of interest:**

No significant relationships.

